# Application of the “syndemics” theory to explain unprotected sex and transactional sex: A cross- sectional study in men who have sex with men (MSM), transgender women, and non-MSM in Colombia

**DOI:** 10.7705/biomedica.5082

**Published:** 2020-06-30

**Authors:** Beatriz E. Alvarado, Héctor Fabio Mueses, Jaime Galindo, Jorge Luis Martínez-Cajas

**Affiliations:** 1 Department of Public Health Sciences, Queens University, Kingston, Canada Department of Public Health Sciences Queens University, Kingston Canada; 2 Corporación de Lucha contra el SIDA, Cali, Colombia Corporación de Lucha contra el SIDA Cali Colombia; 3 Department of Medicine, Division of infectious Diseases, Queen’s University, Kingston, Canada Department of Medicine, Division of infectious Diseases Queen’s University Kingston Canada

**Keywords:** “Syndemic”, HIV, unsafe sex, sexual and gender minorities, “sindémico”, VIH, sexo inseguro, minorías sexuales y de género

## Abstract

**Introduction:**

Men who have sex with men (MSM) and transgender women (TW) in Colombia are highly affected by HIV. To improve understanding of the role of HIV risk behaviors in HIV acquisition, we used the syndemic framework, a useful concept to inform prevention efforts.

**Objective:**

To examine the effect of four psychosocial conditions, namely, forced sex, history of childhood sexual abuse, frequent alcohol use, and illicit drug use on unprotected sex and the synergistic effects (“syndemic” effects) of these conditions on HIV risk behavior.

**Materials and methods:**

We enrolled a total of 812 males (54.7% men who have sex with men, MSM; 7.3% transgender women, and 38% non-MSM). The participants were recruited from neighborhoods of low socioeconomic status through free HIV-counseling and-testing campaigns. We performed Poisson regression analysis to test the associations and interactions between the four psychosocial conditions and unprotected sex with regular, occasional, and transactional partners. To test the “syndemic” model, we assessed additive and multiplicative interactions.

**Results:**

The prevalence of any psychosocial condition was 94.9% in transgender women, 60.1% in MSM, and 72.2% in non-MSM. A higher likelihood of transactional sex was associated in MSM (prevalence ratio (PR)=7.41, p<0.001) and non-MSM (PR=2.18, p< 0.001) with three or all four conditions compared to those with one condition. Additive interactions were present for all combinations of psychosocial problems on transactional sex in MSM. No cumulative effect or additive interaction was observed in transgender women.

**Conclusions:**

Our study highlights the need for bundled mental health programs addressing childhood sexual abuse, illicit drug use, and frequent alcohol use with other HIV prevention programs.

## Resumen

**Introducción.:**

Los hombres que tienen sexo con hombres (HSH), y las mujeres transgenero (MT) en Colombia continuan estando a mayor riesgo de VIH. Para entender como los comportamientos se asocian al VIH, se uso la teoria de la sindemia, la cual se ha considerado muy útil en el desarrollo de estrategias de prevención.

**Objetivo.:**

Examinar el efecto de cuatro afecciones psicosociales, a saber: historia de sexo forzado, historia de abuso sexual infantil, consumo frecuente de alcohol y consumo de drogas ilícitas en las relaciones sexuales sin protección, así como los efectos sinérgicos (efectos “sindémicos”) de estas afecciones sobre el comportamiento de riesgo para HIV.

**Materiales y métodos.:**

Se hizo un estudio transversal que incluyó 812 participantes (hombres que tienen sexo con hombres, HSH: 54,7 %; mujeres transgénero: 7,3 % y hombres que no tenían sexo con otros hombres: 38 %). Los participantes se reclutaron en barrios de estratos socioeconómicos bajos a través de campañas gratuitas de asesoramiento y pruebas de HIV. Se hizo un análisis de regresión de Poisson para probar las asociaciones e interacciones entre las cuatro condiciones psicosociales y las relaciones sexuales sin protección con parejas regulares, ocasionales y comerciales. Para probar el modelo “sindémico” se evaluaron las interacciones aditivas y multiplicativas.

**Resultados.:**

La prevalencia de cualquiera de las condiciones psicosociales fue de 94,9 % en mujeres transexuales, de 60,1 % en HSH y de 72,2 % en hombres que no tienen sexo con hombres. Se encontró una mayor probabilidad de tener sexo comercial en los HSH (razón de prevalencia (RP)=7,41, p<0,001) y en los que no tienen sexo con otros hombres (RP=2.18, p<0,001) con tres de las condiciones psicosociales, o con las cuatro, en comparación con aquellos con una sola condición. Las interacciones aditivas se registraron entre todas las combinaciones de problemas psicosociales con el sexo comercial en los HSH. No se observó un efecto acumulativo ni interacciones en mujeres transexuales.

**Conclusiones.:**

El estudio resalta la necesidad de combinar programas de salud mental que aborden el abuso sexual infantil, el abuso de drogas y el consumo frecuente de alcohol con otros programas de prevención del HIV.

Men who have sex with men (MSM) and transgender women experience greater exposure than other populations to psychosocial health problems such as alcohol and drug use, depression, and different types of violence ^1^^,^^2^.

Men who have sex with men and transgender women are the populations most affected by HIV in Colombia ^3^^,^^4^; a high prevalence of illicit drug use, frequent alcohol use, and early initiation of sexual relations have also been reported in these groups ^5^^,^^6^. A high prevalence of HIV-risk behaviors has also been found in heterosexual men of low socioeconomic status ^7^^,^^8^. Illicit drug use and alcohol abuse have been associated with HIV risk behaviors in these populations ^9^. Heterosexual men are usually a neglected group in HIV prevention programs in Colombia and other Latin American countries despite the increasing recognition of their role in the HIV epidemics ^10^^,^^11^.

Studies conducted in MSM and transgender women have shown that adverse psychosocial conditions can cluster or coexist ^2^ and their accumulation (number of exposures as a cumulative count) increases HIV risk and transmission ^12^. Researchers have also tested the interplay between psychosocial health problems on HIV risk. In a recent analysis of MSM in India, Tomori, *et al*., found an interaction of frequent alcohol use and illicit drug use on the risk of syphilis and intimate partner violence and depression on unprotected anal sex ^13^. Chakaprani, *et al.,* also analyzed MSM from India and found both multiplicative and additive interactions of violence and frequent alcohol use on inconsistent condom use ^14^. These findings mean that the presence of both psychosocial factors confers more risk than the presence of one factor or no factors. These complex interactions relate to the notion of “syndemics”, a concept that has become widely used in HIV research and prevention ^12^^,^^15^.

The “syndemics” framework has been developed to study health conditions affecting populations that can be determined by their contextual factors, be them social, economic, or political. This framework highlights how the clustering and interactions of multiple adversities can amplify the negative impact of one or more other health problems ^16^ in a synergistic fashion. In strict terms, a synergistic effect C is larger than the sum of the effects A plus B. This amplification results where co-occurring outcomes (or health conditions) adversely impact one another: e.g., HIV infection is negatively affected by injection drug use and vice versa. Thus, unveiling the co-occurrence of psychosocial adverse conditions and establishing if they have synergistic (“syndemic”) impact on the health of non-MSM, MSM, and transgender women populations become highly important to define the type of interventions and population groups of higher priority ^15^.

To date, no study has approached HIV risk from the “syndemics” perspective in men of diverse gender/sexual orientation in Colombia. Therefore, this study uses the “syndemics” framework to investigate the effects of alcohol misuse, poly-drug use, sexual violence experience, and history of childhood sexual abuse on unprotected sex with three different categories of partners, namely, regular, occasional, and transactional. In transgender women, transactional sex is particularly important in HIV risk acquisition and is related to greater exposure to psychosocial problems ^17^^,^^18^ and their accumulation ^19^. In MSM, the co-occurrence of psychosocial problems on transactional sex has been less explored despite the high risk of HIV among individuals engaged in transactional sex ^20^^,^^21^ and the associations found between experiences of childhood abuse and sexual violence with higher odds of reporting transactional sex ^22^.

We explored the following aspects:

1. Is there an association between the accumulation of psychosocial factors and unprotected sex?
2. Are there synergistic effects of the presence of two “syndemic” conditions on unprotected sex?, and,
3. Are there differences in these associations according to gender/sexual orientation?

We anticipated a higher prevalence of psychosocial factors in MSM and transgender women versus the non-MSM group. However, we hypothesized that the psychosocial factors had negative effects on unprotected sex across groups in either a synergistic or an additive fashion.

## Materials and methods

### Study setting

The study was conducted in Cali, Colombia, a city with a population of over two million people located in southwestern Colombia. Cali is one of the cities with the fastest growing economy in Colombia and it is a key center for rural-urban migration, as well as a receptor of populations displaced by rural violence. A variety of race/ethnic groups populate Cali and approximately 23% are racial minorities (Afro-Colombians), most of whom are concentrated in low and middle-income neighborhoods. By 2016, Cali had high unemployment (15%) and casual employment (35%) rates and a violent death rate of (70/100,000) ^23^.

### Recruitment

Between 2012 and 2015, participants were recruited from individuals who attended a local HIV counseling and testing campaign. This campaign started by approaching community leaders from the targeted populations: MSM, transgender women, and non-MSM, who were asked to refer individuals whom they thought could benefit from HIV testing/counseling. Interested individuals were instructed to attend community sites where HIV counseling and testing were provided at no cost. At these sites, potential participants were invited to join the study. Informed consent was obtained from all individuals who agreed to participate.

This study focused on adult participants who self-identified as MSM, transgender women, and non-MSM. The study activities were conducted at twenty-two community centers, three sex work venues, and one HIV clinic. At each site, the research personnel applied a paper-based survey before providing HIV counseling and testing following a protocol as reported elsewhere ^24^. The survey was anonymous and was applied in a private room by a trained research assistant. The survey typically took 30 minutes to complete and collected data on sociodemographic characteristics, sexual risk behavior, other risky behaviors, HIV testing, history of sexually transmitted infections, and early sexual experience.

### Study measures

*Psychosocial conditions.* 1) History of childhood sexual abuse: We defined the history of childhood sexual abuse as the reporting of sexual activity at age 13 years or younger because in Colombia adults engaging in sexual activity with children younger than 14 years of age commit an abusive sexual act considered a criminal offense ^25^; 2) Recent history of forced sex was defined as any experience of sex without consent with any sexual partner in the last 12 months; 3) Frequent alcohol use was defined as the consumption of five or more alcoholic drinks in one single occasion more than once in a month, and 4) Use of illicit drugs, which was defined as the consumption of any drug in the last six months, injected or inhaled, from a list of seven options including marijuana, cocaine, *basuco* (cocaine-containing paste) poppers, hallucinogens, heroin, amphetamines, or stimulants. In total, 18% of the sample reported the use of one type of drug, 8% reported two drugs, and 5% reported three or more. Thus, drug use was analyzed as no drugs vs. one or more drugs.

*Main outcomes.* 1) Unprotected sex with regular partners was defined as the frequency of condom use (“most of the time”, “sometimes”, or “never”) with a partner of any sex living permanently with the participant; 2) Unprotected sex with transactional partners was defined as sex not always accompanied of condom use with a partner of any sex with whom they exchanged money or other benefits for sex; 3) Unprotected sex with occasional partners was defined as sex not always accompanied of condom use with a partner of any sex who was not regular or transactional. Transactional partners were infrequent among MSM and non-MSM; we analyzed this variable as a dichotomous outcome (having ever vs. never had a transactional partner in the last 12 months).

*Covariates.* Educational level (highest level of education completed), having health insurance, personal income (based on the minimum monthly wage for Colombia, i.e., USD$ 308 at the time of the survey), history of incarceration and homelessness (defined as currently living on the streets). We also asked the participants to self-report their race/ethnicity. The definition of gender/sexuality was based on men’s self-report of MSM, transgender women, or non-MSM (men who reported themselves as heterosexual).

### Statistical analysis

We followed the analytical recommendations from Tsai, *et al.*^26^. First, we explored the relationship of all psychosocial variables with each outcome in one model to test if their effect was independent of the presence of the other. Next, we tested if the ‘accumulation’ of psychosocial conditions was related to each of the outcomes for which we created an index summarizing the number of psychosocial problems experienced by each respondent. This “syndemic” index was analyzed as a dummy variable ^26^ with the reference point of having one psychosocial problem (given the low frequency of zero psychosocial conditions in transgender women). Next, we calculated the interaction effect of two psychosocial conditions on each outcome. We assessed the additive interaction using the relative excess risk due to interaction (RERI) or part of the total effect due to interaction (null value=0) ^27^ and we calculated the multiplicative interactions using the common approach of exposure1*exposure2 on the outcome.

The significance of the additive interactions was obtained using the ICP program in Stata^™^ (StataCorp LP, College Station Texas, USA). A Poisson analysis with robust variance was performed for all analyses ^28^^).^

## Results

The sample included 812 participants of whom 54.7% self-identified as MSM, 7.3% as transgender women, and 38.0% as non-MSM. More than half of the participants were younger than 25 years old, 18% were from racial minorities, 9% had low levels of education, and 64% had low income. Only 5.4% of the sample was homeless and 9% had experienced incarceration. MSM had more favorable social profiles than transgender women and non-MSM participants with higher levels of education, income, and health insurance (table 1). he non-MSM group was more likely to be homeless and experience incarceration than the other groups. Transgender women had the highest mean of accumulation of psychosocial conditions with more than half of the sample reporting drug use and frequent alcohol use (table 1). The non-MSM group was more likely to report unprotected sex with occasional and regular partners. Having commercial partners was highly prevalent among transgender women, 71% compared to 10.5% for the MSM, and 9% for the non-MSM.

**Table 1:** Description of the sample and comparison of social and psychosocial conditions by gender/sexual orientation

	TW n (%)	MSM n (%)	Non-MSM n (%)	p value
Age (years)				
Less than 25	20 (33.9)	236 (53.15)	174 (56.3)	<0.001
25-40	34 (57.6)	163 (36.71)	78 (25.2)	
>40	5 (8.47)	45 (10.1)	57 (18.5)	
Racial minority				
Afrocolombian or indigenous	18 (30.5)	56 (12.6)	82 (26.6)	<0.001
Highest educational attainment				
<11 years	12 (20.3)	10 (2.26)	54 (17.5)	<0.001
Affiliated to health insurance				
No	17 (29.3)	98 (22.4)	44 (14.6)	0.013
Monthly personal income				
Less than 1 minimum salary	36 (61.0)	259 (58.6)	224 (72.7)	<0.001
Homelessness				
Yes	3 (5.4)	6 (1.4)	34 (11.3)	<0.001
History of incarceration				
Yes	0 (0.0)	0 (0.0)	65 (23.6)	<0.001
Psychosocial conditions				
Childhood sexual abuse	31 (52.4)	91 (20.6)	115 (38.5)	< 0.001
Forced sex, yes	4 (6.9)	12 (2.8)	11 (3.6)	0.26
Frequent alcohol use, yes	40 (67.8)	158 (35.7)	112 (36.4)	<0.001
Illicit drug use, any	35 (59.3)	111 (25.3)	98 (32.2)	<0.001
Index of “syndemics”, mean, SD	1.86, 0.79	0.88, 0.88	1.15, 0.93	<0.001
Number of psychosocial factors				< 0.001
0	3 (5.1)	176 (40.5)	83 (28.3)	
1	14 (23.7)	171 (39.3)	122 (41.6)	
2	30 (50.9)	68 (15.6)	63 (21.5)	
3 or 4	12 (20.3)	20 (4.6)	25 (8.5)	
Sexual risk behaviours				
Have regular partners	31 (52.5)	313 (70.5)	221 (71.5)	0.012
Unprotected sex with regular partners	21 (65.6)	212 (66.8)	197 (87.9)	<0.000
Have occasional partners	43 (72.8)	310 (69.8)	120 (38.8)	<0.000
Unprotected sex with occasional partners	17 (39.5)	127 (41.7)	81 (68.1)	<0.000
Have commercial partners/Transactional sex	42 (71.2)	47 (10.5)	28 (9.06)	<0.000
Unprotected sex with commercial partners	15 (34.8)	19 (38.7)	18 (50.0)	0.37
Unprotected sex, all partners	36 (61.1)	293 (68.1)	224 (85.8)	<0.000

TW: Transgender women; MSM: Men who have sex with men

Table 2 shows an analysis of the effects of all four psychosocial conditions on unprotected sex and transactional sex. Childhood sexual abuse and illicit drug use in MSM, and forced sex in transgender women were associated with unprotected sex with occasional partners. All four psychosocial conditions in MSM and frequent alcohol use in non-MSM increased the risk of transactional sex.

**Table 2:** Multivariate Poisson regression analysis showing effect of combined effects of psychosocial conditions on unprotected sex and transactional sex

	Unprotected sex with regular partner		Unprotected sex with occasional partners		Transactional sex	
	PR	CI95%	PR	CI95%	PR	CI95%
Transgender women						
Illicit drug use	1.50	0.81-2.78	0.72	0.35-1.49	0.99	0.70-1.40
Childhood sexual abuse	0.68	0.41-1.13	0.66	0.31-1.39	1.10	0.78-1.55
Frequent alcohol use	0.72	0.42-1.23	0.54	0.26-1.11	0.97	0.66-1.42
Forced sex	1.24	0.76-2.02	2.15	1.32-3.50	1.07	0.55-2.09
Men who have sex with men						
Illicit drug use	1.03	0.87-1.22	1.45	1.11-1.90	1.85	1.09-3.13
Childhood sexual abuse	1.14	0.96-1.35	1.32	1.00-1.90	2.45	1.47-4.10
Frequent alcohol use	0.83	0.70-0.99	1.04	0.79-1.36	2.52	1.45-4.40
Force sex	1.01	0.65-1.58	1.24	0.72-2.15	2.32	1.14-4.75
Non men who have sex with men						
Illicit drug use	1.02	0.92-1.14	1.14	0.87-1.50	1.18	0.55-2.55
Childhood sexual abuse	1.08	0.97-1.20	1.18	0.89-1.57	1.54	0.74-3.20
Frequent alcohol use	1.03	0.93-1.15	1.08	0.83-1.41	2.48	1.18-5.21
Forced sex	0.99	0.76-1.29	&		1.81	0.50-6.46

&: sample size did not allow calculations

PR: Prevalence ratio ; CI: Confidence intervals; MSM: men who have sex with men

Table 3 presents the bivariate and multivariate analyses for the “syndemic” index. An increase in the number of psychosocial conditions was associated with greater unprotected sex with occasional partners and greater risk of transactional sex in MSM. In transgender women, the cumulative effect was not significant for any outcome. In the non-MSM, the accumulation index did not reach significance, but there was a trend towards an increase in transactional sex as the number of psychosocial conditions increased.

**Table 3:** Multivariate Poisson regression of the accumulation of psychosocial conditions (syndemic index) on HIV related behaviors

Number of psychosocial conditions	Unprotected sex with regular partner		Unprotected sex with occasional partners		Transactional sex	
	PR univariate; CI 95%	PR^&^; CI95%	PR univariate; CI 95%	PR^&^; CI95%	PR univariate; CI 95%	PR^&^; CI95%
TW						
0	--		2.2; 1.14-4.23		0.93; 0.39-2.24	
1	ref		ref		ref	
2	1.41; 0.73-2.72		0.88; 0.37-2.06		0.98; 0.65-1.48	
3 and 4	1.0; 0.43-2.32		0.60; 0.18-1.94		1.05; 0.66-1.68	
MSM						
0	0.98; 0.82-1.17	0.96; 0.80-1.16	1.03; 0.73-1.44	1.01; 0.71-1.43	0.67; 0.29-1.53	0.73; 0.31-1.71
1	ref	ref	ref	ref	ref	ref
2	1.04; 0.84-1.28	1.08;0.88-1.33	1.57; 1.11-2.22	1.58; 1.12-2.24	2.32; 1.11-4.83	2.44; 1.18-5.04
3 and 4	0.78; 0.48-1.28	0.76; 0.46-1.25	2.11; 1.45-3.06	2.10*;1.42-3.09	7.89; 4.19-14.88	7.41;3.79-14.46
Non MSM						
0	0.89; 0.77-1.04	0.89; 0.76-1.03	0.77; 0.50-1.17	0.73; 0.47-1.11	0.40; 0.12-1.40	0.33;0.10-1.13
1	ref	ref	ref	ref	ref	ref
2	1.05; 0.95-1.16	1.08; 0.98-1.18	1.23; 0.94-1.60	1.23; 0.91-1.65	1.41; 0.60-3.33	1.64; 0.69-3.89
3 and 4	0.95; 0.78-1.16	0.95; 0.77-1.17	0.95; 0.61-1.49	0.86; 0.55-1.35	2.22; 0.84-5.83	2.18;0.91-5.20

& All models adjusted by education, income, race/ethnicity, age, and health insurance. Multivariate models for TW were not done because of the small size of the sample.

PR: Prevalence ratio; TW: Transgender women; MSM: men who have sex with men

Table 4 presents the analysis of interactions for MSM. Combinations with forced sex were not calculated due to the presence of empty cells. If the hypothesis of “syndemics” is true, we would observe a greater risk of unprotected sex (or commercial sex) with the presence of two psychosocial conditions, a significant positive RERI, and/or a significant multiplicative interaction. For unprotected sex with regular partners, most of the RERI showed a negative sign but none of the interactions examined were significant. For unprotected sex with occasional partners, the three combinations tested were significant as shown by the PR values in table 4, but only the combination of childhood sexual abuse with alcohol abuse showed a significant multiplicative interaction. For engagement in transactional sex, all three combinations were significant with PR greater than 6 and interactions were significant in an additive scale with RERIs greater than or equal to 4 (table 4).

**Table 4:** Poisson regressions showing the combined effect of two psychosocial conditions on the three outcomes in MSM: Additive and multiplicative interactions, testing of syndemics

Psychosocial conditions		Unprotected sex with occasional partners/ PR (CI95%)	Transactional sex/ PR (CI95%)	Unprotected sex with regular partner/ PR (CI95%)
Childhood sexual abuse	Frequent alcohol use			
No	No	ref	ref	ref
Yes	No	1.05 (0.69-1.60)	1.95 (0.71-5.29)	1.18(0.98-1.43)
No	Yes	0.86 (0.59-1.23)	2.18 (1.02-4.64)	0.86 (0.71-1.05)
Yes	Yes	1.67 (1.22-2.27)	7.6 (3.88-14.89)	0.93 (0.69-1.26)
RERI		0.76 (0.13-1.38)	4.47 (0.46;8.48)	-0.11 (-0.48;0.24)
Multiplicative interaction, p value		0.036	0.33	0.62
Childhood sexual abuse	Illicit drug use			
No	No	ref	ref	ref
Yes	No	1.42 (0.98-2.06)	1.83 (0.80-4.19)	1.21 (1.00-1.48)
No	Yes	1.55 (1.10-2.17)	1.38 (0.59-3.17)	1.08 (0.88-1.32)
Yes	Yes	1.88 (1.31-2.68)	6.15 (3.37-11.22)	1.06 (0.79-1.42)
RERI		-0.10 (-0.94;0.74)	3.94 (0.71-7.17)	-0.24 (-0.65-0.17)
Multiplicative interaction, p value		0.55	0.13	0.24
Illicit drug use	Frequent alcohol use			
No	No	ref	ref	ref
Yes	No	1.36 (0.94-1.94)	1.38 (0.50-3.77)	1.11 (0.92-1.34)
No	Yes	0.97 (0.67-1.40)	2.10 (0.98-4.44)	0.88 (0.72-1.08)
Yes	Yes	1.78 (1.28-2.48)	6.62 (3.39-12.9)	0.84 (0.62-1.13)
RERI		0.45 (-0.22;1.12)	4.14 (0.71-7.57)	-0.16 (-0.51;0.18)
Multiplicative interaction, p value		0.27	0.17	0.39

* significant p<0.05

Table 5 presents the analysis of interactions for non-MSM. There was no clear trend to an increased risk of unprotected sex with combinations of psychosocial conditions. The combination of childhood sexual abuse and frequent alcohol increased the risk of transactional sex (PR=4.2) and the RERI was positive, although non-significant. None of the multiplicative interactions were significant.

In transgender women, none of the interactions were significant (data not shown).

**Table 5:** Poisson regressions showing the combined effect of two psychosocial conditions on the three outcomes in non-MSM: Additive and multiplicative interactions, testing of syndemics

Psychosocial conditions		Unprotected sex with occasional partners/ PR (CI95%)	Transactional sex/ PR (CI95%)	Unprotected sex with regular partner/ PR (CI95%)
Childhood sexual abuse	Frequent alcohol use			
No	No	ref		
Yes	No	1.50 (1.04-2.25)	1.62 (0.51-5.13)	1.17 (1.03-1.32)
No	Yes	1.38 (0.94-2.03)	2.59 (0.90-7.44)	1.12 (0.97-1.28)
Yes	Yes	1.22 (0.76-1.96)	4.32 (1.46-12.77)	1.06 (0.88-1.28)
RERI		-0.66 (-1.45;0.12)	1.10 (-2.40;4.61)	-0.22 (-0.46;0.01)
Multiplicative interaction, p value		0.058	0.97	0.05
Childhood sexual abuse	Illicit drug use			
No	No	ref	ref	ref
Yes	No	1.02 (0.67-1.53)	1.33 (0.50-3.51)	1.07 (0.94-1.22)
No	Yes	1.06 (0.69-1.62)	1.28 (0.42-3.98)	1.03 (0.87-1.21)
Yes	Yes	1.36 (1.02-1.81)	1.89 (0.76-4.71)	1.09 (0.96-1.24)
RERI		0.28 (-0.30;0.86)	0.27 (-1.81;2.36)	-0.01 (-0.23;0.20)
Multiplicative interaction, p value		0.42	0.89	0.89
Illicit drug use	Frequent alcohol use			
No	No	ref	ref	ref
Yes	No	1.48 (1.08-2.01)	2.33 (0.78-6.92)	1.06 (0.94-1.21)
No	Yes	1.20 (0.82-1.73)	3.21 (1.21-8.49)	1.05 (0.92-1.19)
Yes	Yes	1.22 (0.84-1.78)	3.37 (1.14-9.90)	1.05 (0.90-1.22)
RERI		-0.45 (-1.09;0.19)	-1.17(-5.12;2.78)	-0.06 (-0.28; 0.15)
Multiplicative interaction, p value		0.14	0.27	0.53

* significant p<0.05

### Discussion

Our study helps better understand the effects of accumulation of and the interactions between psychosocial conditions on unprotected sex and transactional sex in a diverse sample from Colombia. For the first time in a sample of MSM, transgender women, and non-MSM from Colombia, we tested the “syndemics” framework using 1) the traditional analytic approach of adding the presence of psychosocial conditions and examined their effects on HIV risk behaviors, and 2) two analytical approaches to assess interactions of combinations of psychosocial conditions. Our results highlight the differences across the sample that have important implications for future interventions to address the HIV epidemic in Colombia, as summarized below.

### Findings in MSM

Our findings add to the existing research demonstrating that psychosocial conditions increase the possibility of engaging in HIV risk behaviors in MSM ^3^^,^^29^. Drug use was highly frequent in the sample related to unprotected and transactional sex. The importance of illicit drug use in driving the epidemic of HIV in MSM is well established and our study reinforces these findings ^30^. The role of early life adversities, particularly violent experiences, its high frequency, and its effects on unprotected and transactional sex in MSM in our sample have also been reported in Colombia and other populations ^22^^,^^31^. Forced sex, on the contrary, was reported by a low proportion of the sample with no differences between the groups; it was not related to unprotected sex but was more likely to be present in those who engaged in transactional sex similarly to reports from other authors ^22^^,^^32^. Alcohol abuse has been related to less protected sex in other studies including some from Colombia ^6^^,^^33^ and in our sample, it was related to greater engagement in transactional sex among MSM.

Given the importance of all psychosocial conditions on outcomes in MSM, it is not surprising that the accumulation of psychosocial conditions increased the risk of unprotected sex with occasional partners, which is consistent with most of the published literature in these populations ^34^^,^^35^. The risk of engagement in transactional sex also increases with the accumulation of psychosocial conditions, a novel finding of our study for MSM populations. Approximately 10% of MSM have engaged in transactional sex, consistent with other estimations in Latin American MSM (7%) ^22^.

Of special relevance is the finding of synergistic (or “syndemic”) effects of combinations of psychosocial conditions on unprotected sex and engagement in transactional sex. The risk of unprotected sex with occasional partners increases when both CSA and frequent alcohol use occur together. Chakapri, *et al*. also reported multiplicative interaction effects of the experience of violence and alcohol use on unprotected sex in MSM in India ^14^. We also found synergistic effects for all combinations with childhood sexual abuse, drug use, and frequent alcohol use on transactional sex. We are unaware of other studies reporting similar findings. The fact that some of these psychosocial conditions act synergistically in increasing risky behaviors suggests that even a single intervention, in this case addressing childhood sexual abuse, could greatly reduce engagement in unprotected sex and transactional sex in MSM, even if the other factors are not considered ^15^. The “syndemics” framework claims that clustering and interaction between psychosocial conditions are tied to structural factors. Our findings, then, should be understood as a consequence of structural violence (lack of legal protection) ^36^, social inequities (i.e., unemployment, poverty) ^22^, and minority stress (greater exposure to discrimination and prejudice) ^37^ suffered by MSM populations.

From a public health perspective, our findings emphasize the harmful effects of sexual abuse during childhood ^31^, a critical period for the development of sexual and other health behavior preferences that, if adverse, as is the case of abuse, could lead to higher alcohol abuse, illicit drug use, and violence. This and other experiences of violence throughout life should be addressed and prevented by public health programs and child protection policies. If others confirm the synergistic effects found in our study, acting on experiences of abuse and violence could help prevent HIV cases through their effects in reducing unprotected and transactional sex.

### Findings in transgender women

In our sample of transgender women, 62% engaged in unprotected sex, a figure consistent with another report in a larger Colombian sample ^38^. Childhood sexual abuse, illicit drug use, and alcohol misuse were reported frequently by transgender women, factors that have been related to greater HIV risk behaviors in transgender women elsewhere ^19^^,^^39^. We found only one factor, forced sex, to be related to unprotected sex. Transgender women could have less power to negotiate condom use in abusive relationships. Having transactional partners was also frequently reported (71%) and, in this case, none of the psychosocial conditions was related to this behavior. Our findings contrast with those of other studies in transgender women from North America, India, and Jamaica ^19^^,^^35^^,^^40^ relating the accumulation of psychosocial adversities to greater HIV risk behaviors.

Two factors may have contributed to this lack of results in the direction expected. One is the small sample size. Recruitment of transgender women has always been difficult in Cali as the level of trust in health organizations is poor there (Héctor Mueses, personal communication). We have used community leaders to reach transgender women but even with their enthusiastic assistance, our recruitment was low. In another sample of Latin American transgender women, sample sizes have been over 100 participants while our sample size was 59 ^39^. The other aspect is related to the psychosocial experiences inquired in our survey. Ours was an exploratory study conducted in the context of HIV testing/counseling campaigns. Hence, we felt that in this scenario it was not feasible to inquire more in detail about violence, discrimination, or mental health conditions. In future studies, it would be important to focus specifically on mental health problems, criminalization, social exclusion, and violence displacement as additional psychosocial problems driving HIV risk among transgender women in Colombia ^2^^,^^18^^,^^41^.

### Findings in non-MSM populations

In the case of non-MSM, this study reached a high-risk population not usually targeted for HIV testing or other prevention campaigns. This population lives in poverty, is poorly educated, and one-third of them are racial minorities (Afro-Colombians and aboriginals). Approximately 68% engage in occasional relations with no condom use, the accumulation of psychosocial conditions was even higher than among MSM and apparently associated with higher engagement in transactional sex. As non-MSM could serve as a bridge for HIV and sexually transmitted infection transmission to heterosexual women or bisexual men ^11^, we suggest that non-MSM with low socioeconomic status should be also prioritized for HIV prevention interventions ^11^. It seems that targeting alcohol abuse would be an effective strategy to reduce the risk of unprotected sex in non-MSM.

### Strengths and limitations

Our study is one of the few using the “syndemics” theory to compare HIV risk in groups of diverse gender and sexual preference ^42^. The regular collection of data on HIV and risk behaviors among high-risk populations is not done in Colombia ^43^. The lack of surveillance of behavioral data among high-risk groups may prevent the optimal development and implementation of public health interventions. Our group has taken this initiative and our results point out important implications for the development of HIV prevention programs. We recognize that this study has limitations. The sample was not probabilistic and estimates may not be generalized; participants were a self-selected sample likely based on their perception of risk, knowledge, and experiences of stigma and discrimination related to HIV testing. The MSM sample was captured through leaders of the MSM community and may reflect a selected group with more favorable psychosocial conditions. Our estimates are, however, close to those found using respondent-driven sampling methods with larger samples in Colombia ^3^.

Unfortunately, our sample was underpowered to compare experiences of transgender women and larger samples will be needed for this purpose. Also, our analyses rely on cross-sectional data. In this regard, there is a need for longitudinal studies and intervention trials that allow testing the temporality of psychosocial condition effects, mediation effects, and interactions on the incidence of HIV and risk behaviors ^44^.

### Implications of the results

From a research perspective, our study strongly argues that the “syndemics” theory is a more suitable approach to test the synergistic effects of psychosocial conditions on HIV risk given the multifactorial and complex nature of this problem. Expanding the range and quality of the psychosocial conditions assessed and identifying populations at risk of HIV through drug and alcohol-related venues is also required. Our findings revealed the importance of identifying and addressing mental health (illicit drug use, alcohol use) among MSM, transgender women, and non-MSM ^31^. Interventions using community-based or empowerment theories that have been effective in other populations could be adapted in Colombia and other Latin American countries with similar problems. 
